# West Nile Virus in Europe: Epidemiology, Vector Ecology, Environmental Drivers, and the Role of Equine Sentinel Surveillance in a One Health Framework

**DOI:** 10.3390/pathogens15030298

**Published:** 2026-03-10

**Authors:** Paula Nistor, Livia Stanga, Vlad Iorgoni, Razvan Grigore Cojocaru, Alexandru Gligor, Alexandru Ciresan, Bogdan Florea, Vlad Cocioba, Ionica Iancu, Horia Iorgoni, Cristian Zaha, Cosmin Horatiu Maris, Viorel Herman

**Affiliations:** 1 Department of Infectious Diseases and Preventive Medicine, Faculty of Veterinary Medicine, University of Life Sciences “King Mihai I” from Timişoara, 300645 Timişoara, Romania; paula.nistor@usvt.ro (P.N.); vlad.iorgoni@usvt.ro (V.I.); alexandru.gligor@usvt.ro (A.G.); ionica.iancu@usvt.ro (I.I.); viorel.herman@fmvt.ro (V.H.); 2 Discipline of Microbiology, Faculty of Medicine, “Victor Babes” University of Medicine and Pharmacy, Eftimie Murgu Square 2, 300041 Timişoara, Romania; 3 Department of Surgery, Faculty of Veterinary Medicine, University of Life Sciences “King Mihai I” from Timişoara, 300645 Timişoara, Romania; razvan.cojocaru@usvt.ro (R.G.C.); alexandru.ciresan@usvt.ro (A.C.); cristian.zaha@usvt.ro (C.Z.); 4 Department of Internal Medicine, University of Life Sciences “King Mihai I” from Timişoara, 300645 Timişoara, Romania; bogdan-alexandru.florea.fmv@usvt.ro; 5 Department of Animal Husbandry, University of Life Sciences “King Mihai I” from Timişoara, 300645 Timişoara, Romania; vlad-mihai.cocioba.fmv@usvt.ro; 6 Faculty of Physical Education and Sport, West University of Timișoara, 300223 Timișoara, Romania; horia.iorgoni@e-uvt.ro; 7 Department of Forestry, Faculty of Engineering and Applied Technologies, University of Life Sciences “King Mihai I” from Timișoara, 300645 Timișoara, Romania; cosmin.maris@usvt.ro; 8 Academy of Romanian Scientists (AOSR), Splaiul Independenței 54, 050094 Bucharest, Romania

**Keywords:** West Nile virus, Europe, epidemiology, *Culex*, environmental risk factors, equine, One Health

## Abstract

West Nile virus (WNV) is a mosquito-borne flavivirus that remains an important public and veterinary health concern across Europe. Periodic outbreaks affecting humans, horses, and wildlife highlight the complex ecological interactions underlying viral circulation. This narrative review aims to synthesize current knowledge regarding WNV epidemiology, transmission dynamics, and surveillance strategies in Europe, with particular attention to the Romanian context. Available surveillance data indicate recurrent seasonal transmission in several European regions; however, reported case numbers may be influenced by differences in diagnostic capacity, reporting practices, and surveillance intensity among countries. Recent studies suggest that environmental variability, vector adaptation, and host community composition play important roles in shaping regional transmission risk, although the relative contribution of these factors remains incompletely quantified. Despite expanding surveillance networks and One Health initiatives, important knowledge gaps persist regarding the integration of environmental risk indicators, vector ecology, and operational preparedness into coherent risk-assessment frameworks. This review therefore examines current epidemiological patterns, evaluates surveillance approaches, and discusses emerging drivers of WNV transmission in Europe. As a narrative synthesis based on published literature and surveillance reports, this review is subject to limitations related to heterogeneity in available data and differences in national reporting systems. Nevertheless, a clearer understanding of these interacting factors may support improved surveillance strategies and more adaptive public health responses to future WNV transmission events. Reported surveillance data should be interpreted cautiously, as differences in national surveillance intensity, diagnostic capacity, and reporting frameworks across Europe may influence notified case numbers. Consequently, reported outbreaks do not necessarily reflect proportional differences in transmission intensity.

## 1. Introduction

West Nile virus (WNV) is an arthropod-borne flavivirus transmitted by mosquitoes that causes West Nile fever and neuroinvasive disease in humans and animals [1]. WNV is an enveloped positive-sense single-stranded RNA virus belonging to the genus Flavivirus (family Flaviviridae). Its approximately 11 kb genome encodes a single polyprotein that is cleaved into structural and non-structural proteins required for viral replication and host immune interaction [2]. First identified in Uganda in 1937, West Nile virus is now widely distributed and is regarded as an endemic zoonosis in several European regions, causing recurrent seasonal outbreaks [2]. The virus persists in an enzootic bird–mosquito transmission cycle in which wild birds act as amplifying hosts and ornithophilic *Culex* mosquitoes serve as vectors transmitting the virus between avian species [3]. In humans, infection is asymptomatic in most cases; symptomatic infections typically manifest as an acute febrile illness characterized by fever, headache, and myalgia, while fewer than 1% progress to neuroinvasive disease, including meningitis, encephalitis, or acute flaccid paralysis, particularly in elderly or immunocompromised individuals. Humans and horses are incidentally infected through mosquito bites but, as dead-end hosts, do not develop sufficient viremia to sustain further transmission [4]. Although WNV has circulated in Europe for decades, its re-emergence since the 1990s has significantly reshaped Europe’s epidemiological landscape. The 1996 Bucharest epidemic (~393 confirmed neuroinvasive cases) demonstrated the potential for urban outbreaks in temperate Europe [4,5,6,7]. In the following years, endemic transmission became established in southern and eastern Europe and gradually expanded westward and northward. The detection of WNV lineage 2 in Hungary in 2004, previously confined to sub-Saharan Africa, and its subsequent dissemination across central and southern Europe marked a major shift in the European viral landscape. Lineages 1 and 2 now co-circulate across southern and central Europe with confirmed overwintering capacity [4].

During the past decade, WNV outbreaks have intensified in humans and equines alike. The 2018 season marked the largest epidemic on record, with 1503 human cases and 285 outbreaks among equines reported in the European Union during the transmission season (see Table 1) [8]. Italy, Greece, and Romania accounted for most cases [9]. Continued viral circulation in 2022–2023 confirmed ongoing endemic circulation, consistent with environmental and climatic conditions that may favour vector proliferation, although surveillance intensity and reporting practices may also influence observed trends [4,10]. The *Culex pipiens* complex constitutes the main vector group in Europe, abundant in both urban and rural habitats and capable of bridging avian and mammalian hosts [4,9]. Other species such as *Culex modestus* and some *Aedes* spp. also carry WNV, though their regional importance varies [11]. Favourable transmission conditions have been associated with climatic patterns such as wet springs followed by hot, dry summers, combined with land-use changes including irrigated agriculture and wetland fragmentation [12,13,14,15]. Climatic variables may influence WNV transmission through identifiable biological mechanisms. Higher ambient temperatures accelerate mosquito development, increase biting frequency, and shorten the extrinsic incubation period of the virus within the vector. Milder winters may enhance overwintering survival of infected mosquitoes, while specific rainfall–drought sequences can alter larval habitat availability and concentrate avian hosts and vectors around limited water sources. However, most European evidence remains based on observational associations rather than controlled mechanistic studies, and effect sizes vary substantially between regions [13,16,17]. Apparent expansion of WNV activity in Europe may reflect multiple interacting processes beyond climatic suitability alone, including improvements in surveillance systems, increased diagnostic awareness, land-use modification, urban mosquito adaptation, and changes in avian host communities. Therefore, increases in reported cases should not be interpreted solely as evidence of increased viral transmission. From a veterinary perspective, WNV is an important cause of equine neurologic disease in endemic regions. Affected horses may show fever, ataxia, muscle tremors, weakness, or paralysis, with case-fatality rates that may reach 20–40% among unvaccinated animals [18]. Although horses do not contribute to viral transmission [4], their exposure mirrors human infection risk, making them valuable sentinel indicators for early detection. Continuous equine surveillance, together with monitoring of wild birds and mosquitoes, represents an essential component of Europe’s One Health strategy for prevention and control.

In summary, WNV has re-emerged as a major zoonotic pathogen in Europe, with a widening geographic footprint and recurrent seasonal outbreaks. The relative contribution of climatic change, ecological transformation, viral lineage dynamics, and surveillance intensity to this apparent expansion remains an area of ongoing scientific debate. This review aims to synthesize recent European evidence on the epidemiology, vector ecology, environmental determinants, and One Health surveillance of WNV, with particular emphasis on equine sentinel data and the interaction between climatic, ecological, and surveillance-related factors influencing transmission patterns. Search Strategy and Methods (Structured Narrative Review) A narrative review was conducted covering the period 1 January 2010 to 9 October 2025. The literature search was performed in two rounds (initial search on 15 January 2025 and an updated search on 1 December 2025) in the following databases: PubMed/MEDLINE, Scopus, and Web of Science Core Collection. Grey literature was additionally identified from official institutional sources relevant to WNV surveillance and reporting in Europe (ECDC, EFSA, the European Commission animal disease notification infrastructure, and WOAH).

Search strings. The core PubMed query was:

(“West Nile virus” OR WNV) AND (Europe OR European OR EU OR “European Union” OR EEA) AND (epidemiology OR surveillance OR outbreak* OR “vector competence” OR *Culex* OR “environmental risk” OR climate OR temperature OR rainfall OR drought OR “land use” OR equine OR horse*)

Equivalent queries were adapted for Scopus and Web of Science using database-specific syntax.

Eligibility criteria. We included (i) peer-reviewed studies, reviews, and systematic reviews addressing human and/or animal WNV epidemiology in Europe; (ii) studies on European mosquito vectors (distribution, ecology, competence, insecticide resistance); (iii) studies on environmental or climatic drivers of WNV transmission; (iv) official surveillance outputs and situation updates with clearly defined geographic coverage (EU/EEA vs. EU-neighbouring countries/WHO-Europe); and (v) equine clinical disease, sero-epidemiology, vaccination, and sentinel surveillance in Europe. We excluded non-European primary datasets (unless used strictly for methodological or comparative context), non-scholarly materials, and duplicate reports of the same dataset (the most complete/updated version was retained).

Screening and selection. Records retrieved from each database were exported and deduplicated. Titles and abstracts were screened for relevance, followed by full-text assessment against the eligibility criteria. This study follows a structured narrative review approach. No PRISMA-based study selection workflow or formal risk-of-bias assessment was performed. Evidence was synthesized qualitatively, prioritizing European datasets with clearly described methodology and geographic scope.

Data handling and synthesis. For surveillance numbers, we prioritized official datasets and reports (ECDC surveillance outputs for human cases; European Commission animal disease notifications for equines/birds; EFSA/ECDC joint updates). When numeric values differed across reports, we reported the figure together with the exact reporting system and geographic scope (EU/EEA vs. EU-neighbouring countries) and avoided mixing definitions.

## 2. Epidemiology of WNV in Europe

WNV is recognized as a pathogen with both endemic and epidemic activity in Europe, producing recurrent seasonal outbreaks affecting humans, horses, and birds each year [10]. Serological data indicate that WNV has circulated at low levels since the mid-20th century, but the first large epidemic occurred in Romania in 1996, with approximately 400 confirmed human neuroinvasive cases [10,18]. This event marked a turning point for Europe, demonstrating that WNV can trigger urban epidemics in temperate European regions with notable fatality rates [10]. Subsequent years brought recurrent but localized outbreaks across Southern and Eastern Europe, confirming its capacity for long-term persistence and seasonal recurrence [10,18].

### 2.1. Early Emergence (1990s–2000s)

During the late 1990s and early 2000s, WNV expanded from sporadic detections to widespread regional activity. In 1998, an outbreak of equine encephalitis in Tuscany, Italy, caused by lineage 1, resulted in 14 neurological cases in horses and a 38% herd seroprevalence [4]. Two years later, WNV re-emerged in southern France (Camargue region), with 76 equine cases and 21 fatalities [19]. During this period, regions such as southern Russia, the Balkans, and Central Europe reported new detections of WNV. In 2004, a divergent lineage 2 strain, previously confined to sub-Saharan Africa, was identified in a Hungarian northern goshawk (Accipiter gentilis), triggering widespread outbreaks across central, eastern, and southern Europe [20]. Greece experienced its first major epidemic in 2010, with over 250 human cases (197 of which were neuroinvasive) and 17 equine cases, caused by lineage 2 [21]. By the early 2010s, WNV transmission had become firmly established as endemic in several European countries, including Italy, Greece, Spain, Serbia, Romania, and Hungary [10].

### 2.2. Geographic Expansion and Climatic Influence (2010–2020)

Between 2010 and 2020, WNV transitioned from an emerging pathogen to a stable seasonal threat. It is now endemic in the Mediterranean Basin and the Black Sea region, with sporadic outbreaks in central and western Europe [10]. The countries persistently affected include Italy, Greece, Spain, Serbia, Romania, and Hungary [10]. Warm summers, extensive wetlands, and high densities of *Culex* mosquitoes support viral amplification [16]. Exceptionally hot summers, most notably in 2018, produced Europe’s largest recorded epidemic, with 1503 human cases reported in the European Union during the transmission season [7,8].

Northern and Atlantic European countries, historically considered low-risk, have recently reported autochthonous WNV cases. Germany detected its first equine and avian WNV infections in 2018, followed by human cases in 2019 [2,21]. The Netherlands recorded its first locally acquired human and avian WNV infections in 2020 [10]. These detections illustrate the progressive northward expansion of WNV, consistent with increasing climatic suitability and changing vector ecology *Culex pipiens* and *Culex modestus* are now established in central and northern Europe, suggesting overwintering and sustained local transmission [2,22,23]. By 2023, over 20 European countries had reported WNV in humans, horses, or wildlife [21,24]. However, notified case numbers depend strongly on national surveillance strategies, diagnostic access, and reporting obligations. Consequently, inter-country comparisons based solely on reported case counts should be interpreted cautiously and may not directly reflect underlying transmission intensity. Countries reporting WNV activity include Austria, Germany, Portugal, Czechia, Slovakia, Slovenia, Croatia, Albania, and North Macedonia. In the United Kingdom, WNV has been considered a potential emerging risk; however, no autochthonous human or equine infections have been confirmed to date. No indigenous human infections have been reported in Scandinavian countries [8,25].

### 2.3. Equine Epidemiology and Impact

Horses serve both as sentinel hosts and as clinically susceptible species. Most infections are subclinical, but 10–20% of infected animals develop neurological disease [1,4]. Clinical signs include ataxia, muscle tremors, and limb weakness; severe cases may progress to paralysis or recumbency, with a 20–50% case fatality rate [4,21]. The 2010 Greek epidemic reflected these trends, with approximately 30% mortality [21]. Serological studies confirm widespread subclinical exposure, with reported seroprevalence ranging from 10% to 30% in horses from Spain and Italy [4].

Equine outbreaks have increased in both frequency and geographic extent over the past decade. Year-to-year increases in equine outbreaks likely reflect both true changes in transmission intensity and changes in surveillance intensity and reporting practices across countries [10,24,26]. Coordinated One Health surveillance, integrating equine, avian, and vector monitoring, remains the cornerstone of European WNV control [4,17]. Key milestones in European WNV epizootics and their equine impact are summarized in Table 1.

**Table 1:** Major European Epizootics of WNV (1996–2023), with Affected Regions and Equine Impact. Human case counts are included for context where notable.

Year	Affected Region(s)	Outbreaks Among Equine/Equine Cases
1996	Romania (Bucharest and Danube Plain)	First major European WNV epidemic; ~393 human neuroinvasive cases; equine data not reported [1].
1998	Italy (Tuscany)	14 confirmed equine encephalitis cases (38% seroprevalence); lineage 1 identified [12].
2000	France (Camargue, Hérault)	76 equine WNF cases (21 fatalities); 3 asymptomatic human infections [21].
2010	Greece (Central Macedonia)	17 equine cases; ~262 human infections (197 neuroinvasive) due to lineage 2 [12,22].
2018	Italy, Greece, Serbia, SE Europe	1503 human cases in the European Union; 285 outbreaks among equine [9,10]
2020	Spain (Andalusia), Italy, Greece	Notable WNV activity in Western Mediterranean; outbreaks reported in humans and equines.
2022	Italy, Greece, Romania, Hungary, others	101 outbreaks among equines (EU/EEA); 1133 human cases (EU/EEA) and 1340 cases in EU/EEA and neighbouring countries combined [12].
2023	France, Spain, Central Europe	153 equine outbreaks in seven countries (↑51% vs. 2022); 728 reported human cases (709 locally acquired); the increase may partly reflect enhanced surveillance and reporting changes implemented from 2022 onwards [27].

Reported outbreak numbers represent surveillance notifications and should not be interpreted as direct measures of transmission intensity.

### 2.4. Summary

The European WNV landscape is defined by an expanding geographic range, recurrent seasonal peaks, and multi-host transmission involving birds, equines, and humans [1,10]. The Eastern Mediterranean, Balkans, and Po Valley remain hotspots, while Western Europe is sporadically affected. Recent evidence from northern and western Europe indicates further northward ecological expansion into areas that were previously low-incidence, supporting the need for strengthened surveillance in newly affected regions. Climatic warming, wetland ecology, and anthropogenic land-use change continue to expand the virus’s ecological niche [10]. Integrated One Health surveillance, linking veterinary, entomological, and public health data, remains essential for timely detection, risk assessment, and prevention of large-scale outbreaks [4].

## 3. Vectors of WNV in Europe

Mosquitoes of the genus *Culex* are the primary vectors of WNV in Europe. Among them, the *Culex pipiens* complex, *Culex modestus*, and *Culex torrentium* are the most relevant for maintaining the enzootic cycle and facilitating bridge transmission to mammals, including horses. Other culicid species contribute sporadically and generally have limited epidemiological significance. Understanding their ecology, host preference, and insecticide susceptibility is essential for assessing the risk of equine exposure and improving vector control [12,16,17].

### 3.1. Culex pipiens Complex

The *Culex pipiens* complex is the most widespread mosquito group in Europe and displays remarkable ecological plasticity [28]. It comprises two main ecotypes, *pipiens* (ornithophilic) and *molestus* (mammalophilic and subterranean), with hybrids capable of bridging transmission between birds and mammals. Hybrid populations, common in peri-urban areas, feed on both birds and mammals, making them highly efficient bridge vectors [29,30].

These mosquitoes breed in stagnant water in diverse habitats, from natural marshes and river margins to artificial containers, drains, and barrels [2,30]. Adults are typically active from late spring through early autumn; females overwinter in sheltered sites [22,30,31]. Natural WNV infections have been reported throughout southern and central Europe, and the seasonal peak in *Culex pipiens* abundance coincides with late-summer equine and human outbreaks, confirming its key role in viral spillover [32,33].

### 3.2. Urban Adaptation and Transmission Dynamics

Urban environments provide stable breeding habitats (underground water systems, basements, drainage networks) that favour *Culex pipiens molestus* and hybrid forms. Urban heat island effects may prolong seasonal activity and enhance overwintering survival. Hybridization between ornithophilic and mammalophilic biotypes increases host-bridging potential, facilitating spillover from avian reservoirs to humans and horses. Consequently, urban adaptation represents not merely a habitat shift but a functional change in transmission dynamics, particularly in periurban interfaces where equine facilities coexist with dense human populations [24,32,33].

### 3.3. Culex modestus

*Culex modestus* is a highly efficient bridge vector closely associated with rural wetlands, irrigation canals, and rice fields [12,34,35,36,37,38,39,40,41,42,43,44,45]. Larvae develop in permanent vegetated waters, and adults are active from summer to early autumn, sometimes overwintering as adults [12]. Unlike the mainly ornithophilic *Cx. pipiens pipiens*, *Cx. modestus* feeds readily on both birds and mammals [35,36,37]. Blood-meal analyses indicate frequent feeding on waterfowl, cattle, and horses, facilitating transmission from avian reservoirs to equines and humans [36].

Field investigations in France, Spain, Italy, and Greece have repeatedly detected WNV-infected *Cx. modestus* during outbreaks. In agricultural areas near marshes or rice paddies, this species represents a major vector-related risk factor for grazing horses during summer [37].

### 3.4. Culex torrentium

*Culex torrentium*, a sibling species of *Cx. pipiens*, is predominant in northern and central Europe, especially in cooler climatic regions. It breeds in similar aquatic environments but is more strictly ornithophilic [38,39,40]. Laboratory studies show that at 24–26 °C, infection and transmission rates can exceed those of *Cx. pipiens*, indicating high competence at moderate temperatures [39].

Although it rarely feeds on mammals, its abundance in northern latitudes supports maintenance of the enzootic cycle. Spillover to equines may occur where *Cx. torrentium* overlaps with mammal-biting species or occasionally feeds on mammals [41,42,43,44]. In northern Europe, *Cx. torrentium* dominates, but its strict ornithophilic behavior limits direct equine exposure compared with *Cx. pipiens/molestus*.

### 3.5. Other Potential Vectors

Additional species occasionally implicated include *Culex perexiguus* in Spain and *Aedes detritus* in coastal wetlands [44,45]. Their contribution appears to be secondary and geographically limited. The invasive *Aedes albopictus*, now established across southern Europe, shows experimental competence for WNV but lacks consistent field evidence of transmission. Accordingly, in most European settings Ae. albopictus is best considered a potential or context-dependent vector unless field detection and epidemiological linkage support a role in local transmission [46,47].

### 3.6. Veterinary Relevance and Comparative Competence

Vector ecology directly determines equine exposure risk. Horses pastured near wetlands, rice fields, or irrigation canals face elevated exposure risk due to *Cx. modestus* and hybrid *Cx. pipiens* populations [48]. Urban and peri-urban stables are mainly exposed to *Cx. pipiens molestus* and hybrid forms active in man-made habitats such as drains and basements [49]. Seasonal peaks in vector abundance between July and September align with most equine outbreaks [50]. Experimental vector competence does not necessarily translate into epidemiological importance under field conditions, which depend on mosquito abundance, survival, host-feeding behaviour, and environmental context.

Experimental comparisons demonstrate that at 24–26 °C, *Cx. torrentium* exhibits higher infection and transmission rates than *Cx. pipiens* [40,41], whereas *Cx. modestus* acts as the primary bridge vector in irrigated or rice-field landscapes [34,36]. Accurate characterization of local mosquito community composition, temperature-dependent competence, and phenology is essential for optimizing vaccination timing, surveillance design, and vector control [51]. Host-feeding behaviour in European Culex populations is increasingly recognized as plastic rather than fixed. Blood-meal analyses demonstrate seasonal and spatial shifts in host preference, influenced by host availability, temperature, and habitat structure. Mathematical modelling suggests that even moderate increases in mixed avian–mammalian feeding can disproportionately elevate spillover risk by enhancing bridge transmission. Therefore, vector competence alone is insufficient to predict epidemiological impact; host-feeding plasticity and its environmental modulation are critical components of transmission modelling [12,48,52]. The key ecological and veterinary traits of the main European WNV vectors are summarized in Table 2.

### 3.7. Insecticide Resistance and Control Implications

Beyond individual vector competence, transmission risk depends on vectorial capacity, integrating mosquito density, biting rate, survival probability, and duration of viral incubation within the vector. These interacting ecological parameters may vary substantially between regions and seasons.

Insecticide resistance in *Culex pipiens* has been reported in multiple European regions, including southeastern Europe, Italy, France, and Spain, with documented knockdown resistance (kdr) mutations and reduced susceptibility to pyrethroids and organophosphates [12,31,36]. The distribution and intensity of resistance vary geographically and remain unevenly monitored across Europe [47]. Reduced susceptibility limits the effectiveness of adulticidal spraying and larvicidal treatments, necessitating the implementation of integrated pest management strategies combining environmental sanitation, larval habitat reduction, and targeted biological control. Heterogeneous resistance patterns may alter local vectorial capacity by increasing adult survival under control pressure, thereby indirectly influencing transmission dynamics in endemic areas.

For equine health management, resistance-driven persistence of vector populations increases the need for preventive vaccination, regular stable disinsection, and spatial risk assessment in peri-urban areas where *Cx. pipiens molestus* predominates. Evidence of resistance markers in Romania should therefore be interpreted as a regional example; a Europe-wide synthesis requires harmonised resistance monitoring across countries and vector taxa [51].

Overall, transmission intensity depends not only on vector presence but on the interaction between competence, feeding plasticity, urban adaptation, climatic suitability, and insecticide resistance. Integrative approaches combining entomological surveillance, host-feeding analysis, and ecological modelling are necessary to move beyond species-level descriptions toward predictive risk assessment.

## 4. Environmental and Climatic Risk Factors

While climatic and environmental determinants have been introduced in previous sections to contextualize epidemiological trends, the present section provides a structured analytical synthesis of mechanistic drivers underlying transmission variability. Rather than reiterating epidemiological patterns, we focus here on causal pathways linking temperature, hydrology, land use, and urbanization to vectorial capacity, host aggregation, and spillover probability. This distinction aims to separate descriptive outbreak chronology from environmental risk modelling considerations [13,16,28,45].

The transmission dynamics of WNV in Europe are determined by environmental and climatic conditions that influence mosquito abundance, host–vector contact, and viral amplification. Horses kept outdoors in rural and peri-urban environments are directly exposed to these environmental risk factors [12].

### 4.1. Temperature

Ambient temperature governs mosquito development as well as viral replication. Warm summers accelerate larval growth, increase biting frequency, and shorten the extrinsic incubation period [54]. In Spain, equine outbreaks are associated with weekly mean temperatures above 14 °C, peaking around 30–34 °C; each 1 °C increase, with a four-week lag, correlates with an increased equine infection risk [53,54]. Similar patterns have been reported in Italy, Greece, and Central Europe, where anomalously hot summers (e.g., 2018) produced the largest recorded epidemics [13,55]. Projections indicate that ongoing warming will lengthen the transmission season and expand both the northern and altitudinal limits of climatic suitability for WNV transmission [53].

Although higher temperatures are consistently associated with increased WNV transmission metrics, these relationships remain probabilistic rather than deterministic. Temperature influences vector development rates, survival, and viral replication kinetics; however, outbreak occurrence depends on the interaction between climatic suitability, vector abundance, host availability, viral lineage characteristics, and surveillance sensitivity. Consequently, elevated temperatures should be interpreted as facilitating conditions that increase transmission potential rather than as direct causes of outbreaks. Regional heterogeneity in land use, hydrology, and vector control practices further modulates these associations [14,16,29,45].

### 4.2. Rainfall and Drought

Moderate precipitation in late winter and spring enlarges aquatic habitats for *Culex* vectors. In contrast, hot and dry summers concentrate birds and mosquitoes near limited water sources, intensifying transmission [52]. Studies from Greece, Italy, and the Iberian Peninsula confirm that “wet spring–dry summer” sequences precede equine and human outbreaks [13,14]. Heavy rainfall can temporarily reduce vector density by flushing larvae; therefore, both drought and excess precipitation modulat outbreak intensity [56,57].

### 4.3. Landscape and Land Use

Wetlands, river basins, and irrigated farmland consistently overlap with areas of WNV circulation. Rice fields, irrigation canals, and marshes support *Culex modestus* and *Culex pipiens*, key bridge vectors [13,58]. In the Po Valley and Axios Delta, equine outbreaks overlap with vector abundance in irrigated croplands [50,59,60]. During the 2020 epizootic, infections in Spain and Portugal were concentrated in the Guadalquivir and Guadiana basins, highlighting the hydrographic determinants of equine and human exposure [60]. Horses pastured near these ecosystems remain at elevated risk each summer.

### 4.4. Urban and Peri-Urban Settings

*Cx. pipiens molestus* and hybrid forms breed in drains, basements, and artificial containers, allowing populations to persist under mild winter conditions. Peri-urban zones where birds, humans, and equines overlap serve as hotspots for bridge transmission [61,62,63]. gricultural intensification, irrigation, livestock farming, and habitat fragmentation create additional mosquito breeding niches. Surveillance data from Italy and Spain indicate higher equine seroprevalence near irrigated crops and urban–rural interfaces [14,17,64].

### 4.5. Climate Change and Long-Term Trends

Warmer summers, milder winters, and altered rainfall patterns have progressively expanded the ecological suitability for WNV transmission over recent decades [17]. The number of days with meteorological conditions favourable to transmission has increased in Germany, the Netherlands, and the Balkans, corresponding with the observed northward spread of the virus, exemplified by Germany’s first equine and avian detections in 2018 and the Netherlands’ first local human cases in 2020 [65,66]. Projections indicate rising exposure in previously low-risk areas and longer, more intense seasons in endemic regions.

### 4.6. Synthesis

**Figure 1:**
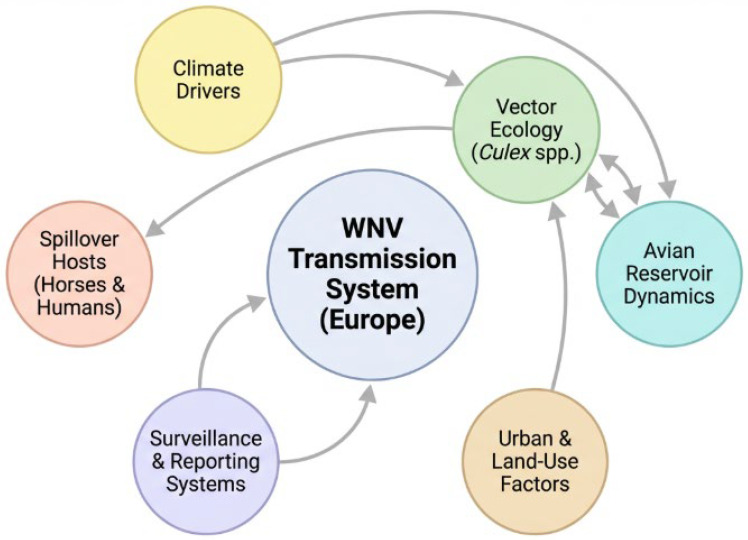
Systems-level conceptual framework illustrating multidirectional interactions among climatic drivers, vector ecology, avian reservoir dynamics, land-use factors, surveillance systems, and spillover hosts in shaping WNV transmission in Europe. Arrows indicate interacting influences rather than simple linear causation.

Associations between climatic variables and WNV transmission reported in European studies are largely correlational and may be influenced by confounding ecological and surveillance-related factors. Drought concentrates hosts and vectors, further enhancing contact rates. A 1 °C increase in weekly mean temperature with a four-week lag has been associated with elevated equine risk in studies from Spain and Italy, with similar associations reported in Greece and Central Europe [13,53,54,55]. Climate change continues to extend both the spatial and temporal windows of WNV transmission, heightening the risk of equine and human outbreaks. These determinants should guide targeted surveillance, vaccination timing, and adaptive vector control in horses and other susceptible species. These relationships between climate, vector ecology, avian reservoirs and spillover to horses and humans are illustrated in Figure 1. Increased testing following initial detections may also amplify reported case numbers, a phenomenon known as surveillance-trigger bias.

## 5. Equine WNV Infections: Clinical Impact and Sentinel Role

WNV is a neurotropic flavivirus, and horses are highly susceptible dead-end hosts. Most equine infections are subclinical, but West Nile neuroinvasive disease (WNND) can be clinically severe [50]. Clinical WNND typically appears 5–10 days post-infection with ataxia and weakness. Common signs include muscle fasciculations, cranial nerve deficits (e.g., facial paralysis, dysphagia), behavioural changes, and, in advanced cases, hindlimb paresis or recumbency [50]. Case fatality among clinically affected horses can be substantial. A Romanian case series reported 25% mortality (1/4) [67], while untreated neuroinvasive cases have been reported to reach up to ~50%; vaccination markedly reduces this risk [67]. Licensed equine vaccines are available in Europe and substantially reduce the risk of clinical neuroinvasive disease; however, vaccination coverage and timing vary between regions and may influence interpretation of serological surveillance data. Product authorisation and official product information are available via the European regulatory network (EMA/European Commission community register). Survivors generally recover within weeks; in the Romanian cohort, three horses recovered fully in 2–4 weeks with supportive therapy. In clinical practice, many affected horses regain normal function within 1–6 months when intensive supportive care is provided [50,68,69].

Most horses exposed to WNV remain asymptomatic. Serological surveys consistently detect antibodies in clinically healthy equines, confirming widespread subclinical infection. A European meta-analysis estimated a pooled seroprevalence of ~8% (95% CI: 5–12%) [70,71,72,73,74]. Large-scale surveys found 5–7% seropositivity in Italy, Spain, Austria, and Czechia, and 15–16% in Poland and Portugal [24,75,76,77,78,79]. In eastern Germany (2020), seroprevalence reached 5.8%, while southwestern France reported ~9% near a 2023 outbreak [70,71,72,73,74]. In western Romania, 8.17% of 306 unvaccinated horses were IgG-positive, indicating local exposure [69]. These values exceed clinical incidence, indicating that more than 90% of infections are subclinical [70].

Interpretation of serological data requires careful consideration. For comparability, seroprevalence estimates should specify the assay used (ELISA vs. PRNT) and report separate IgM/IgG results where available. Vaccination can confound IgG-based seroprevalence estimates; therefore, vaccination history should always be recorded. Detection of IgM antibodies or seroconversion in paired sera improves diagnostic confidence and helps distinguish recent infection from prior exposure. ELISA assays provide broad screening sensitivity, whereas plaque reduction neutralization tests (PRNT) remain the specificity gold standard for confirming true positives, especially in cross-reactive flavivirus regions [7,70].

Equine seroconversion is a sensitive indicator of local transmission. Detection of WNV antibodies in non-traveling horses indicates autochthonous infection. Monitoring equine populations often provides early warning before or concurrent with human or avian cases. Compared with avian mortality surveillance or mosquito RT-PCR monitoring, equine sentinel surveillance provides accessible clinical and serological indicators but may detect circulation later than systematic vector surveillance. Optimal One Health surveillance therefore requires integration of equine, avian, and entomological data streams. In Spain, 2023 surveillance documented intense WNV (and Usutu virus) circulation in horses near newly affected areas, reinforcing their sentinel role [54]. In Germany, equine serosurveys have mapped the northward spread of lineage 2; horses are particularly valuable sentinels in areas where vaccination rates remain low and serology is unconfounded [2]. Across Europe, unvaccinated horses are recommended as sentinel species within One Health surveillance frameworks, complementing human, bird, and mosquito monitoring in endemic zones and flagging transmission in newly affected regions [73,74,75].

### Summary

Equine WNND is relatively uncommon but can be fatal, whereas most infections remain subclinical. Serological surveillance in horses accurately mirrors local virus circulation and often provides the earliest indication of transmission, enabling timely veterinary and vector-control responses within One Health systems. Vaccination should be strategically scheduled before the onset of the local vector season, guided by accumulated degree-day thresholds and historical temperature patterns [76,77,78].

## 6. One Health Perspective and Surveillance in Europe

National preparedness for WNV should be evaluated using measurable operational indicators rather than solely through institutional presence. Key dimensions include surveillance sensitivity (ability to detect low-level circulation), timeliness of case confirmation, integration between human, veterinary, and entomological sectors, laboratory diagnostic capacity, and the responsiveness of vector-control interventions. The existence of multi-agency coordination structures does not necessarily translate into optimal outbreak mitigation unless these components function cohesively and are periodically assessed through performance metrics [2,8,9,11].

WNV surveillance in Europe is based on a One Health framework integrating human, veterinary, and environmental data to enable early outbreak detection and coordinated response. The European Centre for Disease Prevention and Control (ECDC), the European Food Safety Authority (EFSA), and the World Organisation for Animal Health (WOAH) jointly coordinate this multi-sectoral system, compiling notifications of human cases together with animal and entomological reports. Human WNV cases are reported to ECDC through TESSy, and ECDC publishes seasonal and annual surveillance outputs for EU/EEA countries. Regular ECDC–EFSA joint reports, together with expert networks such as VectorNet and EVD-LabNet, ensure that data from all sectors are shared in real time. Interdisciplinary coordination among veterinary, medical, and environmental sectors is essential for effective WNV control in Europe [16,24]. At the European level, ECDC and EFSA jointly publish monthly updates that integrate human and animal surveillance data. National public health institutions, such as the Robert Koch Institute (RKI, Germany), the Hellenic National Public Health Organization (EODY, Greece), and the Istituto Superiore di Sanità (ISS, Italy), oversee human case management and coordinate with veterinary authorities. In affected areas, blood services implement nucleic acid testing (NAT) measures following local detection, in line with national and EU-level risk-management guidance [16,25]. Italy has incorporated blood-safety testing of “substances of human origin” into its One Health strategy. Across the EU/EEA, the scope and intensity of official animal surveillance programs vary substantially between countries [16,25]. Most rely on passive systems based on mandatory human case reporting and voluntary notification of animal morbidity or mortality. Several countries supplement these with active measures, including systematic mosquito trapping, wild-bird sampling, and periodic serological surveys in equines or sentinel species.

Integrated surveillance combines three principal data streams: human, animal, and vector information. Human infections are reported through national networks and compiled by ECDC; neuroinvasive cases trigger alerts and mandatory blood-donor screening. Equine cases serve as environmental sentinels; WNV infections in unvaccinated horses prompt veterinary investigations and are reported via the Animal Disease Information System (ADIS). In Italy, neurologic equines are tested promptly, and results feed directly into national risk assessments and blood-safety protocols. Wild birds are monitored through both passive (dead or moribund birds) and active (capture and sampling) surveillance programs. The Friedrich-Loeffler-Institut in Germany coordinates nationwide avian surveillance for WNV and Usutu virus. Vector monitoring is systematic in endemic regions, particularly Italy and Greece, where *Culex* mosquitoes are trapped biweekly between May and October and screened by RT-PCR. Serological monitoring complements these methods; periodic antibody testing in horses, chickens, or pigeons can detect silent circulation. In the Netherlands, sentinel chickens demonstrated WNV and Usutu seroconversion weeks before human or equine cases, confirming their predictive value [8,79,80,81].

Surveillance structures combine passive clinical reporting with active environmental sampling. Human surveillance is mainly passive but intensified during epidemic peaks. In animals, veterinarians and wildlife centers report suspected cases, while national programs integrate active serological surveys and entomological sampling. Greece conducts annual equine serosurveys and targeted wild-bird monitoring in high-risk zones. Italy’s National Plan for Arbovirus Prevention, Surveillance and Response (PNA 2020–2025) prescribes continuous monitoring of human and animal health, along with systematic mosquito trapping throughout the May–October transmission season. During Italy’s 2022 epidemic, the first detections occurred in mosquito and bird samples weeks before human or equine cases appeared, demonstrating the predictive strength of integrated surveillance.

Several European countries provide models of best practice. Italy pioneered comprehensive One Health WNV surveillance, launching its first national plan in 2010 and consolidating it under PNA 2020–2025 [8,82,83]. The program mandates cross-sector data sharing, so detection in any compartment, human, animal, or vector, immediately triggers action in the others. In the endemic Veneto region, avian and mosquito detections consistently precede human cases, enabling timely vector control and donor screening. Greece maintains a well-established integrated system coordinated by EODY in collaboration with the Ministry of Agriculture, combining human case reporting, serological testing of sentinel horses, clinical follow-up of nearby equines, and both active and passive bird surveillance. Germany, where WNV emerged in 2018, developed a robust One Health network linking RKI’s human case reporting with the Friedrich-Loeffler-Institut’s avian and mosquito programs. Blood donor screening is mandated each summer, and national databases document both autochthonous and imported infections. In Romania, publicly available information indicates established human surveillance systems, whereas systematic equine sentinel surveillance is less consistently described in the scientific literature. Differences between countries should be interpreted cautiously, as surveillance organization, funding, and reporting transparency vary substantially across Europe [77,78,79,80,84].

Key features of national One Health WNV surveillance systems in selected European countries are summarized in Table 3.

Europe’s One Health surveillance architecture thus combines passive clinical reporting with active veterinary and environmental monitoring to produce a unified view of viral activity. Data from horses, birds, mosquitoes, and humans are continuously synthesised so that any positive detection, such as a virus-positive mosquito pool or infected equines, triggers enhanced human surveillance and vector control. Because most human infections are asymptomatic, reliance solely on clinical data would delay outbreak recognition. Real-time exchange among veterinary, entomological, and medical databases remains crucial for guiding interventions, including equine vaccination, vector management, and blood-donor screening.

Despite the expansion of integrated surveillance systems across Europe, formal cost-effectiveness evaluations of WNV prevention strategies remain limited and heterogeneous between countries. The economic efficiency of active vector surveillance, equine sentinel serology, and routine blood-donor nucleic acid testing depends strongly on local incidence, baseline transmission intensity, and healthcare system capacity. In high-incidence regions, early vector detection and targeted blood screening may prevent costly neuroinvasive cases and transfusion-transmitted infections; however, in low-incidence settings, maintaining intensive surveillance infrastructures may generate substantial operational expenditure relative to detected cases. Systematic comparative analyses integrating epidemiological modelling with economic assessment are therefore needed to optimize allocation of public health and veterinary resources under varying regional risk profiles [2,8,11,74].

## 7. Conclusions

WNV transmission in Europe is best understood as the outcome of interacting climatic, ecological, entomological, and surveillance processes rather than as the consequence of a single dominant driver. While increasing climatic suitability may contribute to extended transmission seasons in certain regions, the apparent geographic expansion of WNV likely reflects a combination of vector adaptation, land-use transformation, avian host dynamics, and heterogeneous surveillance intensity. Distinguishing true ecological expansion from surveillance-amplified incidence remains a central analytical challenge.

This review highlights several priority knowledge gaps. First, quantitative integration of vector competence, host-feeding plasticity, and urban adaptation into predictive transmission models remains limited. Second, insecticide resistance monitoring is uneven across Europe and insufficiently linked to epidemiological impact assessment. Third, harmonized genomic surveillance capable of detecting lineage shifts and overwintering dynamics is not yet consistently embedded within national systems. Fourth, cross-country variability in surveillance sensitivity complicates comparative risk assessment and trend interpretation.

Conceptually, future progress requires moving beyond species-level vector descriptions toward systems-based modelling frameworks that integrate climate variability, land use, host ecology, and surveillance artefacts. Embedding equine sentinel data within such integrative platforms may substantially improve early warning capacity. Strengthening interoperable data systems and mechanistic modelling approaches will be critical for anticipating shifts in transmission patterns under evolving ecological conditions.

Rather than framing WNV expansion as climate-driven, a systems-level perspective emphasizing interacting drivers and feedback mechanisms provides a more robust foundation for both research prioritization and public health preparedness in Europe.

## 8. Limitations

This review is limited by heterogeneity among national surveillance systems, under-ascertainment of subclinical infections, and vaccine-induced seropositivity, which may confound equine serology. Variability in data quality and reliance on grey literature for certain outbreak descriptions may also introduce reporting bias. As a narrative review, this study does not include PRISMA-based study selection or formal risk-of-bias assessment, and conclusions rely on qualitative synthesis of heterogeneous evidence.

Although legislative and institutional frameworks are clearly defined, systematic evaluations quantifying surveillance sensitivity, outbreak response time, and the effectiveness of implemented control measures are rarely published in a standardized format. The absence of transparent benchmarking indicators limits objective comparison with other European systems and underscores the need for harmonized preparedness assessment tools at regional level.

## 9. Future Directions

Progress in WNV prevention and control will require interoperable EU-wide surveillance dashboards, standardized genomic thresholds for lineage shift detection, and predictive modelling that integrates environmental and climatic indicators. Incorporating degree-day thresholds to guide vaccination campaigns and blood-donor screening, together with real-time equine seroconversion alerts, will enhance Europe’s capacity for proactive, One Health-based outbreak prevention. Although several candidate vaccines against WNV have undergone clinical evaluation, no human vaccine is currently widely licensed for routine public use, and continued development remains influenced by fluctuating epidemic incidence and feasibility of large-scale efficacy trials.

## Figures and Tables

**Table 2:** Key traits of major European mosquito vectors of West Nile virus.

Vector Species	Typical Breeding Sites	Feeding Preference	Seasonal Activity	Veterinary Relevance
*Cx. pipiens pipiens*	Natural pools, marshes, ditches [14]	Ornithophilic [14,46]	Spring–autumn; females overwinter [14]	Maintains bird–mosquito cycle; limited direct risk to horses [14,46,48]
*Cx. pipiens molestus*	Underground urban water (sewers, basements) [31]	Mammalophilic [14]	Active year-round in warm sites [30]	Bridges virus from birds to mammals in peri-urban stables [46,47,48]
*Cx. pipiens hybrids*	Peri-urban habitats [46,49]	Mixed bird/mammal feeding [14,47]	Summer–autumn [52,53]	Principal bridge vector for equine infection [53,54]
*Cx. modestus*	Rice fields, irrigation canals, wetlands [34]	Birds and mammals [53]	Summer–autumn [34]	Major bridge vector in rural/agricultural settings [55,56,57]
*Cx. torrentium*	Woodland pools, northern wetlands [38]	Ornithophilic [43]	Spring–summer in cooler climates [37]	Maintains enzootic WNV cycles in northern/central Europe [37,38]
Others (*Cx. perexiguus*, *Ae. detritus*, *Ae. albopictus*)	Wetlands, floodplains, urban containers [45]	Variable [46]	Localized [45]	Minor or regionally limited role [45]

**Table 3:** Comparative One Health surveillance features for WNV in selected European countries.

Country	Active Vector RT-PCR Surveillance	Sentinel Equine Serology	Wild-Bird Monitoring	Blood-Donor Screening Trigger	Public Data Access/Dashboard	Key Features/Remarks
Italy	Yes (biweekly May–October, PNA 2020–2025)	Yes (annual regional surveys)	Yes (passive + active)	Automatic trigger after local detection	Yes (ISS regional dashboard)	Model system integrating all sectors; early vector/bird detection precedes human cases.
Greece	Yes (vector trapping May–October)	Yes (annual sentinel horses)	Yes (passive + active)	Triggered at district level	Yes (EODY bulletins)	Stable system with strong cross-sector coordination; equine surveillance predictive.
Germany	Yes (Friedrich-Loeffler-Institut national program)	No (limited sentinel work)	Yes (national program for WNV/USUV)	Mandatory each summer	Yes (RKI/FLI portals)	Emerging system; robust vector and avian monitoring; limited equine component.
Spain	Yes (regional mosquito networks)	Limited (targeted after outbreaks)	Yes (wetland and migratory birds)	Regional trigger	Partial (ECDC–MAP)	Strong reactive capacity; 2020 Andalusian outbreak improved intersectoral data flow.
Romania	Limited (sporadic research-based)	No (passive only)	Yes (ad hoc projects)	Manual activation post-human case	No public dashboard	Needs integration of equine serology and standardized vector monitoring.
Netherlands	Yes (VectorNet collaboration)	No (chickens used as sentinels)	Yes (backyard/zoo birds)	Trigger after human/avian detection	Yes (RIVM map)	Early detection via sentinel chickens; effective early warning system.

## Data Availability

No new data were created or analyzed in this study.

## Abbreviations

The following abbreviations are used in this manuscript:

WNND	West Nile neuroinvasive disease
WNV	West Nile virus
ADIS	Animal Disease Information System
ECDC	European Centre for Disease Prevention and Control
EFSA	European Food Safety Authority
EU/EEA	European Union/European Economic Area
NAT	Nucleic acid testing
TESSy	The European Surveillance System
WOAH	World Organisation for Animal Health
